# Supplementary material for: Uncertainty estimation with prediction-error circuits
Source: Nat Commun. 2025 Mar 28;16:3036. doi: 10.1038/s41467-025-58311-6 (PMC11953419; doi:10.1038/s41467-025-58311-6)
Supplement: Supplementary file 1 — Supplementary Information [file 41467_2025_58311_MOESM1_ESM.pdf]

Supplementary Information

Estimating the uncertainty of sensory inputs with prediction-error circuits

Loreen Hertäg, Katharina A. Wilmes, Claudia Clopath

Contents

A Supplementary Methods **1**
A.1 Network model . . . 1
A.1.1 Prediction-error network model . . . 2
A.1.2 Memory and variance neuron . . . 2
A.1.3 Weighted output . . . 3
A.2 Connectivity . . . 3
A.2.1 Connections between neurons of the PE circuit . . . 3
A.2.2 Connections between the PE circuit and the M neuron . . . 3
A.2.3 Connections between the PE circuit and the V neuron . . . 4
A.3 Inputs . . . 5
A.4 Simulations . . . 5

B Supporting analyses **6**
B.1 Activity of M and V neuron in a simplified model . . . 6
B.2 Impact of PE neurons' gain on estimating mean and variance . . . 7
B.3 Impact of PE neurons' baseline on estimating mean and variance . . . 8
B.4 Modelling the impact of neuromodulators on the sensory weight . . . 9
B.5 Sensory weight and contraction bias . . . 9

C Supplementary Discussion **10**
C.1 Role of interneurons in the PE circuit . . . 10
C.2 Robustness of the results . . . 11
C.3 Alternative network architectures . . . 11
C.4 Model assumptions, simplifications & limitations . . . 12

D Supplementary Figures **14**

A Supplementary Methods

In the following, we describe in more detail the equations for the dynamics of the neurons in the prediction-error circuit, as well as the memory and variance neurons. We then provide the connectivity of the network and the inputs to the neurons for both the mean-field and multi-cell population model. Finally, to ensure reproducibility, we summarize all simulation parameters used for the results shown in the figures.

A.1 Network model

The network model consists of a *lower* and *higher* mean-field PE circuit (Fig. 1). Each PE circuit contains an excitatory nPE neuron and pPE neuron ($N_{nPE} = N_{pPE} = 1$), as well as inhibitory neurons. The inhibitory neurons comprise PV, SOM and VIP neurons ($N_{SOM} = N_{VIP} = 1$, $N_{PV} = 2$). As has been shown in[1], we need two soma-targeting interneurons, one receiving the sensory input and one receiving the prediction, to obtain a perfect nPE and pPE neuron in the same recurrent network. We, therefore, used two PV neurons (as suggested in the original paper). In addition to the core PE circuit, each subnetwork also includes one memory neuron M and one variance neuron V.

In Figure 2 and the corresponding Supplementary Figures, only the lower subnetwork is simulated. In Supplementary Fig. 7, we replaced this lower mean-field PE circuit with a heterogeneous multi-cell population model containing 200 neurons ($N_{SOM} = N_{VIP} = N_{PV} = 20$, 140 excitatory neurons). In Supplementary Fig. 8, the lower PE circuit comprises 1000 copies of the mean-field network to account for selectivity.

In the following, we describe the dynamics of the neurons/compartments in the mean-field network. The equations for the population PE circuit (Supplementary Fig. 7) are directly deduced from the mean-field equations and can also be found in[1]. The equations for the dynamics of the lower and higher subnetworks are identical. Therefore, we do not differentiate between the two in the network equations described below. While the equations governing the dynamics are the same for both subnetworks, the parameters defining these dynamics may vary. The connectivity parameters are provided in Section A.2, and the inputs to the network are detailed in Section A.3.

A.1.1 Prediction-error network model

Each excitatory pyramidal cell (that is, nPE or pPE neuron) is divided into two coupled compartments, representing the soma and the dendrites, respectively. The dynamics of the firing rates of the somatic compartments r_{nE} (nPE neuron) and r_{pE} (pPE neuron) obey[2]

$$
\begin{aligned}
r_{\mathrm{nE}} = [h_{\mathrm{nE}}]_+ \quad \text{with} \quad \tau_E \, \frac{dh_{\mathrm{nE}}}{dt} &= -h_{\mathrm{nE}} + w_{\mathrm{nE}\leftarrow\mathrm{nD}} \cdot r_{\mathrm{nD}} - w_{\mathrm{nE}\leftarrow\mathrm{PV}_1} \cdot r_{\mathrm{PV}_1} - w_{\mathrm{nE}\leftarrow\mathrm{PV}_2} \cdot r_{\mathrm{PV}_2} + I_{\mathrm{nE}}, \\
r_{\mathrm{pE}} = [h_{\mathrm{pE}}]_+ \quad \text{with} \quad \tau_E \, \frac{dh_{\mathrm{pE}}}{dt} &= -h_{\mathrm{pE}} + w_{\mathrm{pE}\leftarrow\mathrm{pD}} \cdot r_{\mathrm{pD}} - w_{\mathrm{pE}\leftarrow\mathrm{PV}_1} \cdot r_{\mathrm{PV}_1} - w_{\mathrm{pE}\leftarrow\mathrm{PV}_2} \cdot r_{\mathrm{PV}_2} + I_{\mathrm{pE}} \qquad (1)
\end{aligned}
$$

where τ_{E} denotes the excitatory rate time constant (τ_{E}=60 ms), the weights $w_{\mathrm{nE}\leftarrow\mathrm{nD}}$ and $w_{\mathrm{pE}\leftarrow\mathrm{pD}}$ describe the connection strength between the dendritic compartment and the soma of the same neuron, and $w_{\mathrm{nE}\leftarrow\mathrm{PV}_1}$, $w_{\mathrm{nE}\leftarrow\mathrm{PV}_2}$, $w_{\mathrm{pE}\leftarrow\mathrm{PV}_1}$ and $w_{\mathrm{pE}\leftarrow\mathrm{PV}_2}$ denote the strength of somatic inhibition from PV neurons (see A.2.1 below). The overall input I_{nE} and I_{pE} comprise the external background and feedforward inputs (see A.3 below). Firing rates are rectified to ensure positivity ($[\bullet]_+$).

The dynamics of the activity of the dendritic compartments r_{nD} (nPE neuron) and r_{pD} (pPE neuron) obey[2]

$$
\begin{aligned}
r_{\mathrm{nD}} = [h_{\mathrm{nD}}]_+ \quad \text{with} \quad \tau_E \, \frac{dh_{\mathrm{nD}}}{dt} &= -\, h_{\mathrm{nD}} + w_{\mathrm{nD}\leftarrow\mathrm{nE}} \cdot r_{\mathrm{nE}} + w_{\mathrm{nD}\leftarrow\mathrm{pE}} \cdot r_{\mathrm{pE}} + w_{\mathrm{nD}\leftarrow\mathrm{M}} \cdot r_{\mathrm{M}} \\
&\quad - w_{\mathrm{nD}\leftarrow\mathrm{SOM}} \cdot r_{\mathrm{SOM}} + I_{\mathrm{nD}}, \\
r_{\mathrm{pD}} = [h_{\mathrm{pD}}]_+ \quad \text{with} \quad \tau_E \, \frac{dh_{\mathrm{pD}}}{dt} &= -\, h_{\mathrm{pD}} + w_{\mathrm{pD}\leftarrow\mathrm{nE}} \cdot r_{\mathrm{pE}} + w_{\mathrm{pD}\leftarrow\mathrm{pE}} \cdot r_{\mathrm{pE}} + w_{\mathrm{pD}\leftarrow\mathrm{M}} \cdot r_{\mathrm{M}} \\
&\quad - w_{\mathrm{pD}\leftarrow\mathrm{SOM}} \cdot r_{\mathrm{SOM}} + I_{\mathrm{pD}}, \qquad (2)
\end{aligned}
$$

where the weights $w_{\mathrm{nD}\leftarrow\mathrm{nE}}$, $w_{\mathrm{nD}\leftarrow\mathrm{pE}}$, $w_{\mathrm{pD}\leftarrow\mathrm{nE}}$ and $w_{\mathrm{pD}\leftarrow\mathrm{pE}}$ denote the recurrent excitatory connections between PCs. $w_{\mathrm{nD}\leftarrow\mathrm{SOM}}$ and $w_{\mathrm{pD}\leftarrow\mathrm{SOM}}$ represent the strength of dendritic inhibition from the SOM neuron. $w_{\mathrm{nD}\leftarrow\mathrm{M}}$ and $w_{\mathrm{pD}\leftarrow\mathrm{M}}$ denote the strength of connection between the memory neuron and the dendrites (see A.2.1 below). The overall inputs I_{nD} and I_{pD} comprise fixed, external background inputs (see A.3 below). We assume that any excess of inhibition in a dendrite does not affect the soma, that is, the dendritic compartment is rectified at zero.

Similarly, the firing rate dynamics of each interneuron is modeled by a rectified, linear differential equation,

$$
\begin{aligned}
r_{\mathrm{X}} = [h_{\mathrm{X}}]_+ \quad \text{with} \quad \tau_I \, \frac{dh_{\mathrm{X}}}{dt} &= -\, h_{\mathrm{X}} + I_{\mathrm{X}} + w_{\mathrm{X}\leftarrow\mathrm{nE}} \cdot r_{\mathrm{nE}} + w_{\mathrm{X}\leftarrow\mathrm{pE}} \cdot r_{\mathrm{pE}} + w_{\mathrm{X}\leftarrow\mathrm{M}} \cdot r_{\mathrm{M}} - w_{\mathrm{X}\leftarrow\mathrm{PV}_1} \cdot r_{\mathrm{PV}_1} \\
&\quad - w_{\mathrm{X}\leftarrow\mathrm{PV}_2} \cdot r_{\mathrm{PV}_2} - w_{\mathrm{X}\leftarrow\mathrm{SOM}} \cdot r_{\mathrm{SOM}} - w_{\mathrm{X}\leftarrow\mathrm{VIP}} \cdot r_{\mathrm{VIP}}, \qquad (3)
\end{aligned}
$$

where r_{X} denotes the firing rate of interneuron type X, and the weight $w_{\mathrm{X}\leftarrow\mathrm{Y}}$ denotes the strength of connection between the presynaptic neuron Y and the postsynaptic neuron X ($X \in \{\mathrm{PV}_1, \mathrm{PV}_2, \mathrm{SOM}, \mathrm{VIP}\}$, $Y \in \{\mathrm{nPE}, \mathrm{pPE}, \mathrm{PV}_1, \mathrm{PV}_2, \mathrm{SOM}, \mathrm{VIP}, \mathrm{M}\}$). Values can be found in the Supplementary Data 1-3 (see A.2.1). The rate time constant τ_I was chosen to resemble a fast $\mathrm{GABA_A}$ time constant, and set to 2 ms for all interneuron types included. The overall input I_{X} comprises fixed, external background inputs and feedforward sensory inputs (see A.3 below).

A.1.2 Memory and variance neuron

In addition to the core PE circuit, we simulate a memory neuron M and a variance neuron V in each subnetwork. The memory neuron is modeled as a perfect integrator, receiving synapses from both the nPE and pPE neuron,

$$
\tau_E \cdot \frac{dr_{\mathrm{M}}}{dt} = w_{\mathrm{M}\leftarrow\mathrm{pE}} \cdot r_{\mathrm{pE}} - w_{\mathrm{M}\leftarrow\mathrm{nE}} \cdot r_{\mathrm{nE}}. \qquad (4)
$$

$w_{\mathrm{M}\leftarrow\mathrm{pE}}$ denotes the connection strength between the pPE neuron and the memory neuron, and $w_{\mathrm{M}\leftarrow\mathrm{nE}}$ denotes the connection strength between the nPE neuron and the memory neuron (see A.2.2, Eqs. 8, with parameters also provided in Supplementary Tab. 1 for the mean-field model and in Supplementary Fig. 0 for the population model). The time constant $\tau_E = 60$ ms. Please note that although the time constants for the lower and higher M neurons are identical, their *effective* time constants differ due to differences in the weights connecting the PE neurons with the M neurons (the effective time constant of the higher subnetwork is between 4 and 64 times greater than that of the lower subnetwork, see A.2.2).

The dynamics of the variance neuron obeys a non-linear differential equation with leak term,

$$\tau_V \cdot \frac{dr_\mathrm{V}}{dt} = -r_\mathrm{V} + \left(w_{\mathrm{V}\leftarrow\mathrm{pE}} \cdot r_\mathrm{pE} + w_{\mathrm{V}\leftarrow\mathrm{nE}} \cdot r_\mathrm{nE}\right)^2. \tag{5}$$

The weight $w_{\mathrm{V}\leftarrow\mathrm{pE}}$ represents the connection strength between the pPE neuron and the variance neuron, while $w_{\mathrm{V}\leftarrow\mathrm{nE}}$ denotes the connection strength between the nPE neuron and the variance neuron (see A.2.3, with Eqs. 9 and 10, and parameters also provided in Supplementary Tab. 1 for the mean-field model and in Supplementary Fig. 0 for the population model). To ensure that the V neuron encodes the variance, we chose a quadratic activation function. In Supplementary Fig. 11, we used a linear activation function to investigate the impact of the input-output transfer function on the weighting of sensory inputs and predictions. The time constant τ_V was $5\,s$ in the mean-field model, $2\,s$ in the heterogeneous multi-cell population model (Supplementary Fig. 7), and $0.5\,s$ in the network model with selectivity (Supplementary Fig. 8).

A.1.3 Weighted output

The weighted output $r_\mathrm{out}(t)$ is a linear combination of the current sensory input $s(t)$ and the activity of the memory neuron, $r_\mathrm{M}(t)$, inspired by Bayesian multisensory integration (see, e.g.[3]),

$$r_\mathrm{out}(t) = \alpha \cdot s(t) + (1-\alpha) \cdot r_\mathrm{M}(t). \tag{6}$$

How strongly either the sensory input or the prediction thereof contributes to the output is denoted by the sensory weight α,

$$\begin{aligned} \alpha &= \frac{r_{\mathrm{V}_\mathrm{lower}}^{-1}}{r_{\mathrm{V}_\mathrm{lower}}^{-1} + r_{\mathrm{V}_\mathrm{higher}}^{-1}} \\ &= \left(1 + \frac{r_{\mathrm{V}_\mathrm{lower}}}{r_{\mathrm{V}_\mathrm{higher}}}\right)^{-1}. \end{aligned} \tag{7}$$

A.2 Connectivity

A.2.1 Connections between neurons of the PE circuit

The connectivity between neurons of the PE circuit, both for the mean-field and the multi-cell population network, were taken from[1] (the connectivities for the mean-field networks tested are provided in the Supplementary Data 1-3, the connectivity of the multi-cell population model is provided in the Supplementary Data 4). We considered three mean-field networks (see Supplementary Table 2) that differed in terms of the inputs (feedforward vs. feedback) onto the SOM and VIP neurons, and, hence, in their connectivity that established an E/I balance in the excitatory neurons.

A.2.2 Connections between the PE circuit and the M neuron

While the nPE neurons inhibit the M neuron, the pPE neurons excite it. To ensure that the activities of the memory neurons represent the mean of the sensory stimuli in the lower PE circuit and the mean of the prediction in the higher subnetwork, respectively, the net effect of nPE and pPE neurons must cancel in the steady state (see Analysis in B.2). Hence, the weights need to account for the neurons' potentially different gain factors (g_nPE and g_pPE) and the neuron numbers (N_nPE and N_pPE):

$$\begin{aligned} w_{\mathrm{M}\leftarrow\mathrm{nE}} &= \frac{\lambda^\mathrm{loc}}{g_\mathrm{nPE} \cdot N_\mathrm{nPE}} \\ w_{\mathrm{M}\leftarrow\mathrm{pE}} &= \frac{\lambda^\mathrm{loc}}{g_\mathrm{pPE} \cdot N_\mathrm{pPE}} \end{aligned} \tag{8}$$

where λ^{loc} denotes a weight for the lower or higher-order PE circuit, loc $\in \{\mathrm{lower}, \mathrm{higher}\}$. In the lower PE circuit, $\lambda^{\mathrm{lower}} = 3 \cdot 10^{-3}$ for the mean-field model in Fig. 2 and $\lambda^{\mathrm{lower}} = 4.5 \cdot 10^{-2}$ for Figs. 3-5. In the higher PE circuit, $\lambda^{\mathrm{higher}} = 7 \cdot 10^{-4}$.

For the mean-field networks ($N_{\mathrm{nPE}} = N_{\mathrm{pPE}} = 1$), the gain factors g_{nPE} and g_{pPE} are given in Supplementary Table 1. For the multi-cell population network, the gain factors for all PE neurons are shown in Fig. 0 and are given in Supplementary Data 5.

**Network**	**MFN 1**<br>FF → SOM<br>FB → VIP	**MFN 2**<br>FB → SOM<br>FF → VIP	**MFN 3**<br>FF → SOM<br>FF → VIP
nPE	1	1.7	2.5
pPE	1	1.7	2.5

Supplementary Table 1. Gain factors for nPE and pPE neurons in three different mean-field networks (MFN). Each MFN differs with respect to the inputs onto SOM and VIP neurons. The interneurons either receive the feedforward (FF) or feedback (FB) input. All numbers are rounded to the first digit.

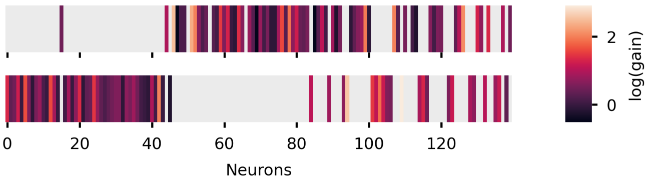

Supplementary Figure 0. Gain factors of nPE and pPE neurons in the multi-cell population model. The logarithm of the gain factors of nPE (top) and pPE (bottom) neurons in the multi-cell population model from [1]. The network contains 67 nPE neurons and 66 pPE neurons. The remaining excitatory neurons were not classified as PE neurons and were not connected to the M neuron.

The memory neuron M connects to the post-synaptic neurons X in the PE circuit with the connection strength $w_{\mathrm{X}\leftarrow\mathrm{M}} = 1$, if a connection exists, $w_{\mathrm{X}\leftarrow\mathrm{M}} = 0$ otherwise. In all mean-field networks and the multi-cell population network, the dendrites of nPE and pPE neurons and one of the two (populations of) PV neurons receive connections from the memory neuron. Furthermore, we assume that the M neuron does not excite the soma of PCs. Whether the SOM or VIP neurons are the target of the feedback projections depend on the specific mean-field network (see Supplementary Table 2). In the multi-cell population model, 30% of the SOM neurons and 70% of the VIP neurons receive input from the memory neuron.

**Network**	**MFN 1**<br>FF → SOM<br>FB → VIP	**MFN 2**<br>FB → SOM<br>FF → VIP	**MFN 3**<br>FF → SOM<br>FF → VIP
SOM	0	1	0
VIP	1	0	0

Supplementary Table 2. $w_{\mathrm{X}\leftarrow\mathrm{M}}$ for the post-synaptic SOM and VIP neurons in all three mean-field networks considered.

A.2.3 Connections between the PE circuit and the V neuron

Both nPE and pPE neurons excite the V neuron. To ensure that the activity of the V neuron represents the variance of the input (see Analysis in B.2), the weights must account for differences in the gains (g_{nPE} and g_{pPE}, see Supplementary Table 1 and Fig. 0) and numbers (N_{nPE} and N_{pPE}) of the PE neurons,

$$\begin{aligned} w_{\mathrm{V}\leftarrow\mathrm{nE}} &= \frac{\theta}{g_{\mathrm{nPE}} \cdot N_{\mathrm{nPE}}} \\ w_{\mathrm{V}\leftarrow\mathrm{pE}} &= \frac{\theta}{g_{\mathrm{pPE}} \cdot N_{\mathrm{pPE}}}. \end{aligned} \tag{9}$$

The factor θ denotes the unscaled weight, and can be chosen to compensate for potential deviations from the definition of the variance as a result of a quadratic activation function. In the mean-field

network, $\theta = 1$ because nPE and pPE neuron activity is mutually exclusive, and, hence, the cross-term nPE $\cdot$ pPE would be zero (under the assumption that they have a negligible baseline activity). This is also true for the multi-cell population model. Each PE neuron receives the same feedforward stimulus and contributes only a small (scaled) fraction to the overall PE (adding up to the same PE used in the mean-field network).

However, in Supplementary Fig. 8, each mean-field network receives a different stimulus s_i drawn from a distribution at time t. While the stimuli below the mean of the distribution activate the nPE neurons (each located in a different mean-field network), the stimuli above the mean of the distribution activate the pPE neurons (each located in a different mean-field network). Because the V neuron first sums all the contributions from the PE neurons before applying the non-linearity, its steady state activity is similar to the squared sum of the *averaged* nPE and *averaged* pPE neuron activity. In this case, θ must be chosen such that deviations from the true variance can be mitigated or fully corrected. The true θ depends on the distribution at hand. In our simulations, we used a uniform distribution $U(a, b)$, in which case θ can be derived from

$$\begin{aligned}\left(\sum_i r_{\mathrm{nE,i}} + \sum_i r_{\mathrm{pE,i}}\right)^2 &\overset{(i)}{=} \left(\frac{\theta}{N}\sum_{s_i \geq \mu}^{N/2}(s_i - \mu) + \frac{\theta}{N}\sum_{s_i \leq \mu}^{N/2}(\mu - s_i)\right)^2 \\ &= \frac{\theta^2}{4}\left(\frac{2}{N}\sum_{s_i \geq \mu}^{N/2} s_i - \frac{2}{N}\sum_{s_i \leq \mu}^{N/2} s_i\right)^2 \\ &\overset{(ii)}{=} \frac{\theta^2}{4}\left(\frac{b+\mu}{2} - \frac{\mu + a}{2}\right)^2 = \frac{(b-a)^2}{16}\cdot\theta^2, \qquad (10)\end{aligned}$$

where we assumed that (i) the number of nPE and pPE neurons is equal, and (ii) this number goes to infinity. Comparing eq. (10) with the equation for the variance of a uniform distribution, $\frac{(b-a)^2}{12}$, we get $\theta = \frac{2}{\sqrt{3}}$.

A.3 Inputs

Each neuron (type) receives an overall input I_i,

$$I_i = I_i^{BL} + w_i \cdot I_i^{FF}$$

where I_i^{FF} denotes a feedforward input and I_i^{BL} represents an external background input that ensures reasonable baseline firing rates in the absence of sensory inputs and predictions thereof. In the case of the mean-field network, these inputs were set such that the baseline firing rates are $r_{\mathrm{pE}} = r_{\mathrm{pD}} = r_{\mathrm{nE}} = r_{\mathrm{nD}} = 0\,s^{-1}$ and $r_{\mathrm{P}} = r_{\mathrm{S}} = r_{\mathrm{V}} = 4\,s^{-1}$. In the case of the multi-cell population network, we set the external inputs of all neuron types to $5\,s^{-1}$, while the background inputs to the dendrites were computed such that the dendrites are inactive during baseline.

The feedforward input is either the direct sensory input s for the lower PE circuit, or the activity of the M neuron, r_{M}, for the higher PE circuit. In general, for the three mean-field networks tested, we chose $w_i = 1 - w_{\mathrm{X}\leftarrow\mathrm{M}}$ (see Supplementary Table 2). In the multi-cell population network, 70% of the SOM neurons and 30% of the VIP neurons receive the feedforward input.

A.4 Simulations

All simulations were performed in customized Python code written by LH. Source code to reproduce the simulations, analyses and figures will be available after publication at `https://github.com/lhertaeg/weighted_sensory_prediction`. Differential equations were numerically integrated using a 2nd-order Runge-Kutta method. Neurons were initialized with $r = 0/s$.

The qualitative results were fairly robust to the choice of the simulation parameters and are here stated merely to ensure the reproducibility of all figures. However, we note that we made use of PE circuits that had been trained on steady state inputs[1]. Hence, we must simulate the network long enough to ensure that the PE neurons reach their steady state. Moreover, the lower-level M neuron must evolve faster than the higher-level M neuron as indicated in Supplementary Fig. 11. Finally, the time constant of the V neurons must be of the same magnitude as the trial duration.

In Supplementary Table 3, we give all SI figure-specific parameters not directly visible or mentioned in the figures and captions (see Table 1 for the main figures). In a trial, N_{in} constant values were drawn

from a normal distribution $N(\mu_{\mathrm{in}}, \sigma^2_{\mathrm{in}})$, each N_{step} time steps long. The stimulus mean was drawn from an uniform distribution, $U(a, b)$, with mean μ_{in} and variance $\sigma^2_{\mathrm{trial}}$.

Supplementary Table 3. Parameters used to stimulate the network for the Supplementary figures. To increase readability, we do not include units for the parameters. All units can be deduced from the equations. N_{in}: number of values each stimulus is composed of, N_{step}: number of consecutive time steps each value is presented, trial duration is given by $N_{\mathrm{in}} \cdot N_{\mathrm{step}}$, $\mu_{\mathrm{in}}/\sigma^2_{\mathrm{in}}$: mean/variance of the normal distribution from which the stimulus values are drawn, (a, b): lower and upper bound of the uniform distribution μ_{in} is drawn from in each trial, $\sigma^2_{\mathrm{trial}}$: standard deviation of the uniform distribution μ_{in} is drawn from in each trial. − indicates that parameter/s is/are not used in the simulation.

Fig S		# trials	N_{in}	N_{step}	μ_{in}	σ^2_{in}	(a, b) or $\sigma^2_{\mathrm{trial}}$	pert. stgth	note
2		1	200	500	5	4	-	-	-
3		1	200	500	5	4	-	-	#seeds: 20
4	A-D	2	200	500	5	4	-	-	-
5	A,B	1	200	650	5	4	-	-	-
	C	200	10	500	5	1/0/1	0/1/1	-	-
6	col 2,3	1	300	500	5	4	-	-	-
	col 4	150	10	500	5	25	25	-	-
7	B	1	200	500	5	4	-	-	dt=0.1,
	C-E	1	200	500	5	4	-	-	#seeds: 10
									$\gamma \in$ N(0,1)
8	B	1	4000	1	$4 \to 6$	$4 \to 4$	-	-	#net: 1000
					$4 \to 4$	$4 \to 6$			
	C	1	4000	1	[3,6]	[3,6]	-	-	#net: 1000
9	B	100	10	500	-	$0 \to 9$	(5,5)	-	switch
						9	$(5,5) \to (0,10)$		after
						$9 \to 0$	(0,10)		50 trials
						0	$(0,10) \to (5,5)$		
10		100	10	500	-	$0 \to 9$	$(0,10) \to (5,5)$	-	see S9
11	A, B	100	10	500	5	[0,9]	$\sigma^2_{\mathrm{trial}} \in$[0,9]	-	-
13		200	10	500	5	5	0	0.5	100 trials
14		200	10	500	20/30	$\mu_{\mathrm{in}} - 14$	(15,25) / (25,35)	-	-
15	A-C	200	10	500	5	0	1	0.5	perturb.
16	A-C	200	10	500	5	1	0	0.5	in the last

B Supporting analyses

We first describe a simplified model and show that the M neuron represents the mean, while the V neuron represents the variance of the feedforward input. We then investigate the impact of the gain and baseline of PE neurons on estimating the mean and variance. Furthermore, we use the simplified model to discuss the effect of neuromodulators in our network. Finally, we reveal the connection between the sensory weight and the contraction bias.

B.1 Activity of M and V neuron in a simplified model

To show that the M neuron encodes the mean, while the V neuron encodes the variance of the feedforward input, we resume a toy model in which the activity of the nPE and pPE neuron is replaced by its ideal output

$$
\begin{aligned}
r_{\mathrm{nE}} &= [r_{\mathrm{M}} - s_{\mathrm{FF}}]_+ \\
r_{\mathrm{pE}} &= [s_{\mathrm{FF}} - r_{\mathrm{M}}]_+
\end{aligned} \tag{11}
$$

with s_{FF} denoting the time-dependent feedforward input. The activity of the M neuron can then be described as

$$\tau_M \cdot \frac{dr_{\mathrm{M}}}{dt} = r_{\mathrm{pE}} - r_{\mathrm{nE}} \tag{12}$$

If $r_{\mathrm{M}} \geq s_{\mathrm{FF}}$, we get

$$\tau_M \cdot \frac{dr_{\mathrm{M}}}{dt} = -r_{\mathrm{nE}} = -r_{\mathrm{M}} + s_{\mathrm{FF}}. \tag{13}$$

If $r_{\mathrm{M}} \leq s_{\mathrm{FF}}$, we also get

$$\tau_M \cdot \frac{dr_{\mathrm{M}}}{dt} = r_{\mathrm{pE}} = -r_{\mathrm{M}} + s_{\mathrm{FF}}.$$

Hence, the activity of r_{M} is given by

$$r_{\mathrm{M}} = \frac{1}{\tau_M} \int_0^t e^{-(t-x)/\tau_M} \cdot s_{\mathrm{FF}}(x)\ dx \tag{14}$$

for zero activity at time $t = 0$. In the limit of $t \to \infty$ (steady state), this is the exponential moving average of the feedforward input, $E(s_{\mathrm{FF}})$.

With the simplified activity of the nPE and pPE neuron, the activity of the V neuron can then be described as

$$\tau_V \cdot \frac{dr_{\mathrm{V}}}{dt} = -r_{\mathrm{V}} + (r_{\mathrm{pE}} + r_{\mathrm{nE}})^2 = -r_{\mathrm{V}} + (r_{\mathrm{M}} - s_{\mathrm{FF}})^2, \tag{15}$$

leading to the time-dependent solution

$$r_{\mathrm{V}} = \frac{1}{\tau_V} \int_0^t e^{-(t-x)/\tau_V} \cdot [r_{\mathrm{M}}(x) - s_{\mathrm{FF}}(x)]^2\ dx. \tag{16}$$

In the limit of $t \to \infty$, r_{V} approaches $E(s_{\mathrm{FF}} - E(s_{\mathrm{FF}}))$.

B.2 Impact of PE neurons' gain on estimating mean and variance

The gains of the PE neurons, if not equal between the nPE and pPE neuron on average, can bias the activity of both the M and V neuron. To show this, we resume the toy model from section B.1.

$$\begin{aligned} g_{\mathrm{pPE}}\ \langle r_{\mathrm{nPE}} \rangle &= g_{\mathrm{nPE}}\ \langle r_{\mathrm{pPE}} \rangle \\ g_{\mathrm{pPE}}\ \langle [s_{\mathrm{FF}} - P]_+ \rangle &= g_{\mathrm{nPE}}\ \langle [P - s_{\mathrm{FF}}]_+ \rangle \\ g_{\mathrm{pPE}} \int_P^\infty (x - P)\ f(x)\ dx &= g_{\mathrm{nPE}} \int_{-\infty}^P (P - x)\ f(x)\ dx. \end{aligned} \tag{17}$$

Here, P denotes the prediction encoded in the M neuron, and $f(x)$ is the distribution of feedforward inputs. In case of a uniform distribution, $f(x) = 1/(b-a)$ for $x \in [a, b]$ and 0 otherwise, we get

$$P = \begin{cases} \frac{a+b}{2} & \text{if } g_{\mathrm{nPE}} = g_{\mathrm{pPE}} = g \\ \frac{g_{\mathrm{pPE}} \cdot b - g_{\mathrm{nPE}} \cdot a + \sqrt{g_{\mathrm{nPE}}\ g_{\mathrm{pPE}}}\ (a-b)}{g_{\mathrm{pPE}} - g_{\mathrm{nPE}}} & \text{otherwise.} \end{cases} \tag{18}$$

Hence, the mean of the feedforward input is overpredicted when $g_{\mathrm{nPE}} < g_{\mathrm{pPE}}$. Similarly, the mean of the feedforward input is underpredicted when $g_{\mathrm{nPE}} > g_{\mathrm{pPE}}$ (Supplementary Fig. 12).

Likewise, the variance is affected by the gain of the nPE and pPE neuron,

$$\begin{aligned} V &= \langle (r_{\mathrm{pPE}} + r_{\mathrm{nPE}})^2 \rangle \overset{(i)}{=} \langle r_{\mathrm{pPE}}^2 \rangle + \langle r_{\mathrm{nPE}}^2 \rangle \\ &= g_{\mathrm{pPE}}^2\ \langle [s_{\mathrm{FF}} - P]_+^2 \rangle + g_{\mathrm{nPE}}^2\ \langle [P - s_{\mathrm{FF}}]_+^2 \rangle, \end{aligned} \tag{19}$$

where we assume (i) that both the nPE and pPE neuron have a zero baseline activity. In case of a uniform distribution, we get

$$V = \frac{g_{\mathrm{pPE}}^2}{b-a} \int_P^b (x-P)^2 \; dx + \frac{g_{\mathrm{nPE}}^2}{b-a} \int_a^P (P-x)^2 \; dx \tag{20}$$
$$= \frac{g_{\mathrm{pPE}}^2}{3} \cdot \frac{(b-P)^3}{b-a} + \frac{g_{\mathrm{nPE}}^2}{3} \cdot \frac{(P-a)^3}{b-a}.$$

Inserting eqs. (18) yields

$$V = \begin{cases} \frac{(b-a)^2}{12} & \text{if } g_{\mathrm{nPE}} = g_{\mathrm{pPE}} = 1 \\ \frac{(b-a)^2}{3\;(g_{\mathrm{pPE}} - g_{\mathrm{nPE}})^3} \cdot \left[g_{\mathrm{nPE}}^2 \cdot (g_{\mathrm{pPE}} - \gamma)^3 - g_{\mathrm{pPE}}^2 \cdot (g_{\mathrm{nPE}} - \gamma)^3 \right] & \text{otherwise.} \end{cases} \tag{21}$$

with $\gamma = \sqrt{g_{\mathrm{nPE}} \; g_{\mathrm{pPE}}}$. Hence, the variance of the feedforward input is overpredicted when $g_{\mathrm{nPE}} > 1$ or $g_{\mathrm{pPE}} > 1$. Similarly, the variance of the feedforward input is underpredicted when $g_{\mathrm{nPE}} < 1$ or $g_{\mathrm{pPE}} < 1$ (Supplementary Fig. 12).

B.3 Impact of PE neurons' baseline on estimating mean and variance

The baselines of the PE neurons, if not equal between the nPE and pPE neuron on average, can bias the activity of both the M and V neuron. By means of the toy model from section B.1, we can write

$$\langle r_{\mathrm{pPE}} \rangle = \langle r_{\mathrm{nPE}} \rangle \tag{22}$$
$$\langle [s_{\mathrm{FF}} - P]_+ + p_0 \rangle = \langle [P - s_{\mathrm{FF}}]_+ + n_0 \rangle$$
$$\int_P^\infty (x-P)\; f(x)\; dx + p_0 \underbrace{\int_a^b f(x)\; dx}_{=1} = \int_{-\infty}^P (P-x)\; f(x)\; dx + n_0 \underbrace{\int_a^b f(x)\; dx}_{=1}.$$

n_0 and p_0 denote the baseline activity of the nPE and pPE neuron, respectively. In case of a uniform distribution (c.f. B.2), we get

$$P = \frac{b+a}{2} + \frac{p_0 - n_0}{b-a}. \tag{23}$$

Thus, the M neuron encodes the true mean of the feedforward input only if $p_0 = n_0$. As a result, the mean is overpredicted if $p_0 > n_0$. Likewise, the mean is underpredicted if $p_0 < n_0$ (see Supplementary Fig. 12).

With non-zero baseline activities, the steady state activity of the V neuron is given by

$$V = \langle (r_{\mathrm{pPE}} + r_{\mathrm{nPE}})^2 \rangle \tag{24}$$
$$= \langle [s_{\mathrm{FF}} - P]_+^2 \rangle + \langle [P - s_{\mathrm{FF}}]_+^2 \rangle + (p_0 + n_0)^2 + 2\;(p_0 + n_0)\;\big(\langle [s_{\mathrm{FF}} - P]_+ \rangle + \langle [P - s_{\mathrm{FF}}]_+ \rangle \big)$$

In case of a uniform distribution $U(a, b)$, this expression yields

$$V = \frac{1}{3\;(b-a)} \left[(b-P)^3 + (P-a)^3 \right] + (p_0 + n_0)^2 + \frac{(p_0 + n_0)}{b-a} \left[(b-P)^2 + (a-P)^2 \right]. \tag{25}$$

Inserting the expression for P (Eq. 23) which is itself a function of the baseline activities, gives

$$V = \frac{(b-a)^2}{12} + \frac{(p_0 - n_0)^2}{(b-a)^2} \left(1 + 2\;\frac{p_0 + n_0}{b-a} \right) + (p_0 + n_0) \left(p_0 + n_0 + \frac{b-a}{2} \right). \tag{26}$$

Thus, for the V neuron to encode the variance unbiased, $n_0 = p_0 = 0$. The variance is overpredicted if either $n_0 > 0$ or $p_0 > 0$ (see Supplementary Fig. 12).

B.4 Modelling the impact of neuromodulators on the sensory weight

We modeled the presence of a neuromodulator by simulating an additive excitatory input onto (groups of) interneurons. These interneurons, in turn, modulate the gain and baseline of PE neurons. As shown in sections B.2 and B.3, changes in the input-output transfer function of the PE neurons may bias the variance estimation in the network, and, hence, the sensory weight. Thus, understanding changes in the sensory weight requires an understanding of whether and how different types of interneurons change the PE neurons.

If a neuromodulator only acts on interneurons of the lower-level subnetwork, the sensory weight changes as a consequence of the modulated firing rates of the lower-level and higher-level V neurons. The lower-level V neuron is directly affected by the changes in the lower-level PE neurons and indirectly affected by changes in the M neuron of the same network. The higher-level V neuron is also affected by a neuromodulator acting in the lower-level subnetwork because the lower-level M neuron projects onto the neurons in the higher-level PE circuit. Hence, if the lower-level M neuron represents a biased mean $\mu \pm \delta\mu$, the variance estimation will be biased as well. This can be seen directly from the definition of the variance,

$$\begin{aligned} V &= \frac{1}{n}\sum_i \left(x_i - (\mu \pm \delta\mu)\right)^2 \\ &= \frac{1}{n}\sum_i \{(x_i-\mu)^2 + \delta\mu^2 \mp 2\,\delta\mu\,(x_i-\mu)\} \\ &= V_{\text{unmod}} + \delta\mu^2 \mp 2\;\delta\mu\left(\frac{1}{n}\sum_i x_i - \mu\right) \\ &= V_{\text{unmod}} + \delta\mu^2 \end{aligned}$$

In contrast, if a neuromodulator only acts on interneurons of the higher-level subnetwork, the sensory weight changes as a consequence of the modulated firing rates of the higher-level V neuron. The higher-level V neuron is directly affected by the changes in the higher-level PE neurons and indirectly affected by changes in the M neuron of the same network.

Together, this suggests that whether the sensory weight decreases, increases, or remains the same in the presence of a neuromodulator depends on several factors:

- Does the neuromodulator act on the lower-level or higher-level subnetwork (that is, local impact), or does the neuromodulator act on both to the same degree (that is, global impact)?
- Which interneuron type/s is/are affected by the neuromodulator? And are these interneurons inhibited or excited by the neuromodulator?
- How are these interneurons embedded in the network, that is, what are the connectivity and the inputs to those neurons?

As a result, different neuromodulators may have the same effect on the sensory weight or the same neuromodulator may have different effects depending on brain area, species, etc..

B.5 Sensory weight and contraction bias

In the simulations, we define the bias as the trial-averaged difference between the weighted output and the true stimulus. For the sake of simplicity, we use r_{out} at the end of a trial, T, as a proxy for the trial average in the subsequent analysis. Hence,

$$\text{bias} = r_{\text{out}}(T) - s. \tag{27}$$

To investigate how the bias depends on the sensory weight and potentially other factors, let us resume a toy model in which we assume that the prediction decays exponentially with time constant τ to a presented constant stimulus value, s,

$$P = P_0 \cdot \mathrm{e}^{-t/\tau_M} + s \cdot \left(1 - \mathrm{e}^{-t/\tau}\right) \tag{28}$$

with P_0 describing the initial value at time $t = 0$. Let us further assume that within a trial with trial duration T, the stimulus value changes n times ($T = n \cdot \Delta t$). The prediction during the presentation of the nth stimulus value can be expressed as

$$P_\mathrm{n} = P_0 \cdot \mathrm{e}^{-\Delta t/\tau_M} + \left(1 - \mathrm{e}^{-\Delta t/\tau_M}\right) \sum_{i=1}^{n} s_i \cdot \mathrm{e}^{-(n-i)\cdot \Delta t/\tau_M}. \tag{29}$$

To obtain an estimate for the prediction at the end of a trial, P_n must be averaged over the stimulus distribution, $\langle P_\mathrm{n} \rangle_s$. For the sake of simplicity, let us assume the stimulus values are drawn from a uniform distribution $U\left(s - \frac{\sigma_\mathrm{S}}{12}, s + \frac{\sigma_\mathrm{S}}{12}\right)$. Moreover, we assume that the initial state, P_0, at the beginning of a new trial is drawn from a uniform distribution $U\left(\mu - \frac{\sigma_\mathrm{P}}{12}, \mu + \frac{\sigma_\mathrm{P}}{12}\right)$. With these assumptions, $\langle P_\mathrm{n} \rangle_s$ is given by

$$\langle P_\mathrm{n} \rangle_s = \mathrm{e}^{-\Delta t/\tau_M} \int_{\mu - \frac{\sigma_\mathrm{P}}{12}}^{\mu + \frac{\sigma_\mathrm{P}}{12}} P_0 \; f(P_0) \; dP_0 + \left(1 - \mathrm{e}^{-\Delta t/\tau_M}\right) \sum_{i=1}^{n} \mathrm{e}^{-(n-i)\cdot \Delta t/\tau_M} \int_{s - \frac{\sigma_\mathrm{S}}{12}}^{s + \frac{\sigma_\mathrm{S}}{12}} x \; f(x) \; dx. \tag{30}$$

Solving the integrals yield

$$\langle P_\mathrm{n} \rangle_s = \mu \cdot \mathrm{e}^{-T/\tau_M} + \left(1 - \mathrm{e}^{-\Delta t/\tau_M}\right) \sum_{i=1}^{n} \mathrm{e}^{-(n-i)\cdot \Delta t/\tau_M} \cdot s. \tag{31}$$

Making use of the geometric series, the expression simplifies to

$$\langle P_\mathrm{n} \rangle_s = \mu \cdot \mathrm{e}^{-T/\tau_M} + \left(1 - \mathrm{e}^{-T/\tau_M}\right) \cdot s.$$

Inserting the expression in the equation for the weighted output yields

$$r_\mathrm{out} = \left[\alpha_\mathrm{S} \; \mathrm{e}^{-T/\tau_M} + \left(1 - \mathrm{e}^{-T/\tau_M}\right)\right] \cdot s + (1 - \alpha_\mathrm{S}) \; \mathrm{e}^{-T/\tau_M} \; \mu.$$

Hence, the bias in our toy model can be expressed by

$$\mathrm{bias} = (1 - \alpha_\mathrm{S}) \cdot \mathrm{e}^{-T/\tau_M} \cdot (\mu - s).$$

The absolute slope $(1 - \alpha_\mathrm{S}) \cdot \mathrm{e}^{-T/\tau_M}$ indicates how strong the bias is. It depends on the sensory weight α_S, the trial duration T and time constant τ_M. Please note that the sensory weight is a function of the trial duration itself (see Fig. 3F). However, for illustration purposes, we take α_S to be constant.

In this toy model, if the variance of the prediction is zero (that is, in a prediction-driven input regime), $\alpha_\mathrm{S} \approx 0$, and, consequently, the bias is $\mathrm{e}^{-T/\tau_M} \cdot (\mu - s)$. Thus, the bias is independent of the stimulus variance (see Fig. 5 D).

Likewise, if the variance of the sensory stimulus is zero (that is, in a stimulus-driven input regime), $\alpha_\mathrm{S} \approx 1$, and, consequently, the bias approaches 0 if the neurons reach their steady state. Thus, decreasing or increasing the trial variance does not have an effect on the bias (see Fig. 5 C).

C Supplementary Discussion

C.1 Role of interneurons in the PE circuit

We include three types of inhibitory interneurons in our network: PV, SOM and VIP interneurons. Generally speaking, these interneurons are required to establish nPE and pPE neurons by balancing the multiple pathways the sensory inputs and predictions can take through the network[1]. More precisely, the PV neurons establish an E/I balance at the soma of the excitatory neurons, while the SOM neurons establish an E/I balance at the dendrites of the same neurons. In addition, we include VIP neurons that are known to receive top-down inputs and to provide disinhibition[4–10]. These VIP neurons and more importantly the connections they make with other interneurons are necessary to ensure that the E/I balance required for PE neurons is met not only for fully predicted sensory inputs but also for one of the mismatches (sensory input < prediction, or sensory input > prediction).

In our mean-field network, we include only one cell per interneuron type except for PV neurons which we assumed to be represented by two neurons. The reason for that is that we have previously shown[1]

that only one source of somatic inhibition is not sufficient to give rise to both nPE and pPE neurons in the same network in which the dendrites are balanced for fully predicted sensory inputs and during one of the two mismatch phases (for more information, please see[1]). To account for that, we included two PV neurons, one receiving the sensory inputs while the other one receiving the prediction thereof.

Please note though that other network architectures are possible. For instance, we can develop PE circuits with less inhibitory interneuron types but those networks would require more constraints on the distribution of sensory inputs and predictions among the remaining interneurons. This, however, might not be in line with the rich spectrum of inputs neurons receive in biological networks. Moreover, some of the constraints on the interneuron circuit can be relaxed if we only require nPE and pPE neurons to exhibit an E/I balance for fully predicted sensory inputs and allow them to be over-inhibited in one of the mismatch phases.

C.2 Robustness of the results

We showed that the lower-level subnetwork can correctly estimate the mean and the variance of the sensory inputs for different stimulus implementations (Supplementary Fig. 4), statistics (Fig. 2) and distributions (Supplementary Fig. 3). Moreover, the results hold for a multi-cell population model used to estimate the sensory uncertainty over time (Supplementary Fig. 7) or space (Supplementary Fig. 8). Furthermore, we showed that the baseline firing rate of nPE and pPE neurons must rather be small or canceled by respective interneurons to ensure an unbiased estimation of the sensory uncertainty (Supplementary Fig. 5).

We further investigated how the estimation of the mean and the variance is influenced by connection strengths and neuron properties (Supplementary Fig. 6). Scaling the connections from the PE neurons onto the memory neuron only affects the speed at which the mean is reached (Supplementary Fig. 6B). If the connections from the M neuron are scaled, the mean is encoded in the product of the memory neuron's activity times this connection strength (Supplementary Fig. 6C). If additional arbitrary top-down input targets the neurons in the PE circuit that also receive synapses from the memory neuron, the memory neuron encodes the mean reduced by this top-down input (Supplementary Fig. 6E). In contrast, the V neuron is mainly affected by its time constant and the connection it receives from the PE neurons. While the time constant determines how much the estimated variance fluctuates around the true variance, scaling the connections onto the V neuron biases the variance estimation (Supplementary Fig. 6A & D).

In the full network, these biases manifest in a sensory weight that is slightly pushed towards 0.5 (Supplementary Fig. 6). That is, in a former sensory-driven regime, the dependence on the sensory inputs is slightly weakened. In contrast, in a former prediction-driven regime, the dependence on the sensory inputs is slightly strengthened. Similarly, while other properties like the connectivity between the PE neurons and the M/V neurons can affect the estimation of the mean and the variance, the sensory weight is only slightly affected if the changes occur in both the lower- and the higher-level circuit.

C.3 Alternative network architectures

We focus here on *one* network whose core units are PE circuits. In this network, the nPE and pPE neurons drive both the M and the V neurons, which in turn encode the mean and the variance of the feedforward input (in the lower-level circuit: sensory input, in the higher-level circuit: prediction). This implementation has the advantage, first, of explicit mean and variance representations in excitatory cells, which could be broadcasted to other areas, and second, provides a prediction and an output even in the absence of sensory input. While an exhaustive exploration of different models would be beyond the scope of this work, we will discuss some alternatives in the following.

First of all, we modeled the memory neuron as a perfect integrator. An alternative would be to model it as a leaky integrator with a time constant significantly larger than zero. The leaky memory neuron would directly receive the feedforward input and project to the PE circuit, thereby representing a low-pass filter of the feedforward input. In contrast to our implementation, the memory neuron would be silent without any sensory information. Hence, it can not be interpreted as a "prediction" which should also be available in the absence of sensory evidence. While, in our model, we do need the sensory information to develop a prediction in the first place (here the mean of the sensory input), the input is not necessary after the prediction has been established.

Second, we propose that the activity of PE neurons is utilized to encode uncertainty in a downstream neuron. However, alternative models offer different mechanisms for this process. One such alternative suggests that a neuron could independently compute the squared error between the sensory input and

its mean. Wilmes et al. (2023) demonstrate that a PV neuron with a quadratic activation function can represent variance in its activity without requiring direct connections from PE neurons. In their model, the incoming weights onto the PV neurons learn to store input variability through a local activity-dependent plasticity rule[11]. Moreover, in the model proposed by Wilmes et al. (2023), the PE itself is modulated by uncertainty, a feature absent in our model.

Another alternative involves estimating variance through an error-minimizing learning rule that reduces the discrepancy between the squared magnitude of errors and a variance estimate[12]. This model predicts that the error on the variance estimate is encoded by a third class of error neurons, but it does not predict the existence of an excitatory cell type that represents the variance itself, as our model does. Additionally, Granier et al. (2023) propose that error activity occurs in the apical dendrites of the representation neurons.

Besides these alternatives for modeling the uncertainty, we have recently proposed a network that implements a neuronal contraction bias[1]. The main difference is that in the previous paper, the output neuron received not only the sensory input but also synapses from the nPE and pPE neurons. While the nPE neurons excited the output neuron, the pPE neuron inhibited it. This way, the contraction bias was a direct result of the opposite connectivity between the PE neurons and the output neuron, and the amount of bias was dictated by the connection strength between those neurons. Hence, the bias is independent of the uncertainties of the sensory input or the prediction, and only scales with the absolute difference between both inputs. In contrast, in our current model, we assume that the network output would not receive direct input from the PE neurons but the prediction itself, and that both the sensory input and the prediction is weighted by their reliabilities (Fig. 5).

C.4 Model assumptions, simplifications & limitations

As with any computational model, we simplify certain biological details to maintain the model's simplicity and interpretability. However, those details, while beyond the scope of this study, may be well investigated in future work. For instance, we have simplified the input-output activation function of the neurons. The memory neurons are modeled with a linear tuning curve and a broad dynamic range. This contrasts with typical neurons, which are usually feature-specific and respond to a limited range of inputs. However, homeostatic plasticity can help neurons adjust their sensitivity to inputs, thereby aligning their dynamic range with the statistical properties of the input signals[13,14]. One example is synaptic scaling[13,15], where the strengths of all synapses on a neuron are scaled up or down to maintain overall activity within a target range. Additionally, neurons can adjust their gain, or sensitivity to inputs, based on the statistical properties of the inputs they receive[16].

The sensory input in our work can vary on two timescales: changes in the stimulus caused by noise are faster than those caused by switches in the environment. To account for this, the lower-level memory neuron must operate on a faster timescale than the higher-level memory neuron. To achieve this, the weights from the PE neurons onto the memory neuron are larger for the lower than the higher PE circuit. This assumption is consistent with the observation that time constants increase along the cortical hierarchy[17–19]. While we have set these weights in our network, they are more likely subject to plasticity, allowing them to be learned and adjusted according to the stimulus timescales that can change in real life.

Neuromodulatory systems have been suggested to gate plasticity[20]. In a recent study by Jordan and Keller[21], locus coeruleus (LC) axon activity is shown to correlate with the magnitude of unsigned visuomotor prediction errors. The authors hypothesize that LC output modulates the learning rate at which the internal model evolves[21]. In our model, we do not consider precision-weighted PEs (but see[11,12]). Hence, a sensible extension to our work would be to adjust the weights from PE neurons onto the memory neurons by a function of the stimulus and prediction uncertainty, respectively. This would allow us to compare our results more closely to work showing that ACh and NA can adjust the rate at which new sensory evidence is incorporated when environments change[22,23].

It has been hypothesized that some symptoms in psychiatric diseases may derive from an erroneous uncertainty estimation[24]. For instance, hallucinations may arise from an underestimation of the expectation uncertainty or an overestimation of the sensory uncertainty. Conversely, a fixation on the environment, even when the sensory cues indicate a switch in the environment, may originate from an overestimation of the expectation uncertainty or an underestimation of the sensory uncertainty[24]. Several factors influence the accuracy of the variance estimation, leading to potential over- or underestimation of the true variance. For instance, the limited duration of trials (and thus the stimulus duration) inherently restricts the precision of variance estimation. Moreover, neurons do not operate instantaneously. Their activity upon a stimulus is governed by time constants that introduce transient responses. These ON/OFF responses

impact the variance estimation, especially when the noise levels are high and the stimulus changes rapidly. Additionally, the PE neurons in our network exhibit a small baseline activity, which may result in the overestimation of small variances.

References

1. Hertäg, L. & Clopath, C. Prediction-error neurons in circuits with multiple neuron types: Formation, refinement, and functional implications. *Proceedings of the National Academy of Sciences* **119**, e2115699119 (2022).

2. Wilson, H. R. & Cowan, J. D. Excitatory and inhibitory interactions in localized populations of model neurons. *Biophysical journal* **12**, 1–24 (1972).

3. Pouget, A., Beck, J. M., Ma, W. J. & Latham, P. E. Probabilistic brains: knowns and unknowns. *Nature neuroscience* **16**, 1170–1178 (2013).

4. Pfeffer, C. K., Xue, M., He, M., Huang, Z. J. & Scanziani, M. Inhibition of inhibition in visual cortex: the logic of connections between molecularly distinct interneurons. *Nature neuroscience* **16**, 1068–1076 (2013).

5. Lee, S., Kruglikov, I., Huang, Z. J., Fishell, G. & Rudy, B. A disinhibitory circuit mediates motor integration in the somatosensory cortex. *Nature neuroscience* **16**, 1662–1670 (2013).

6. Pi, H.-J. *et al.* Cortical interneurons that specialize in disinhibitory control. *Nature* **503**, 521–524 (2013).

7. Zhang, S. *et al.* Long-range and local circuits for top-down modulation of visual cortex processing. *science* **345**, 660–665 (2014).

8. Harris, K. D. & Shepherd, G. M. The neocortical circuit: themes and variations. *Nature neuroscience* **18**, 170 (2015).

9. Letzkus, J. J., Wolff, S. B. & Lüthi, A. Disinhibition, a circuit mechanism for associative learning and memory. *Neuron* **88**, 264–276 (2015).

10. Tremblay, R., Lee, S. & Rudy, B. Gabaergic interneurons in the neocortex: from cellular properties to circuits. *Neuron* **91**, 260–292 (2016).

11. Wilmes, K. A., Petrovici, M. A., Sachidhanandam, S. & Senn, W. Uncertainty-modulated prediction errors in cortical microcircuits. *bioRxiv* 2023–05 (2023).

12. Granier, A., Petrovici, M. A., Senn, W. & Wilmes, K. A. Precision estimation and second-order prediction errors in cortical circuits. *arXiv preprint arXiv:2309.16046* (2023).

13. Turrigiano, G. Homeostatic synaptic plasticity: local and global mechanisms for stabilizing neuronal function. *Cold Spring Harbor perspectives in biology* **4**, a005736 (2012).

14. Turrigiano, G. G. The dialectic of hebb and homeostasis. *Philosophical transactions of the royal society B: biological sciences* **372**, 20160258 (2017).

15. Turrigiano, G. G. & Nelson, S. B. Homeostatic plasticity in the developing nervous system. *Nature reviews neuroscience* **5**, 97–107 (2004).

16. Fairhall, A. L., Lewen, G. D., Bialek, W. & de Ruyter van Steveninck, R. R. Efficiency and ambiguity in an adaptive neural code. *Nature* **412**, 787–792 (2001).

17. Murray, J. D. *et al.* A hierarchy of intrinsic timescales across primate cortex. *Nature neuroscience* **17**, 1661–1663 (2014).

18. Chaudhuri, R., Knoblauch, K., Gariel, M.-A., Kennedy, H. & Wang, X.-J. A large-scale circuit mechanism for hierarchical dynamical processing in the primate cortex. *Neuron* **88**, 419–431 (2015).

19. Runyan, C. A., Piasini, E., Panzeri, S. & Harvey, C. D. Distinct timescales of population coding across cortex. *Nature* **548**, 92–96 (2017).

20. Pawlak, V., Wickens, J. R., Kirkwood, A. & Kerr, J. N. Timing is not everything: neuromodulation opens the stdp gate. *Frontiers in synaptic neuroscience* **2**, 146 (2010).

21. Jordan, R. & Keller, G. B. The locus coeruleus broadcasts prediction errors across the cortex to promote sensorimotor plasticity. *Elife* **12**, RP85111 (2023).

22. Marshall, L. *et al.* Pharmacological fingerprints of contextual uncertainty. *PLoS Biology* **14**, e1002575 (2016).

23. Bruckner, R., Heekeren, H. R. & Nassar, M. Understanding learning through uncertainty and bias (2022).

24. Yon, D. & Frith, C. D. Precision and the bayesian brain. *Current Biology* **31**, R1026–R1032 (2021).

D Supplementary Figures

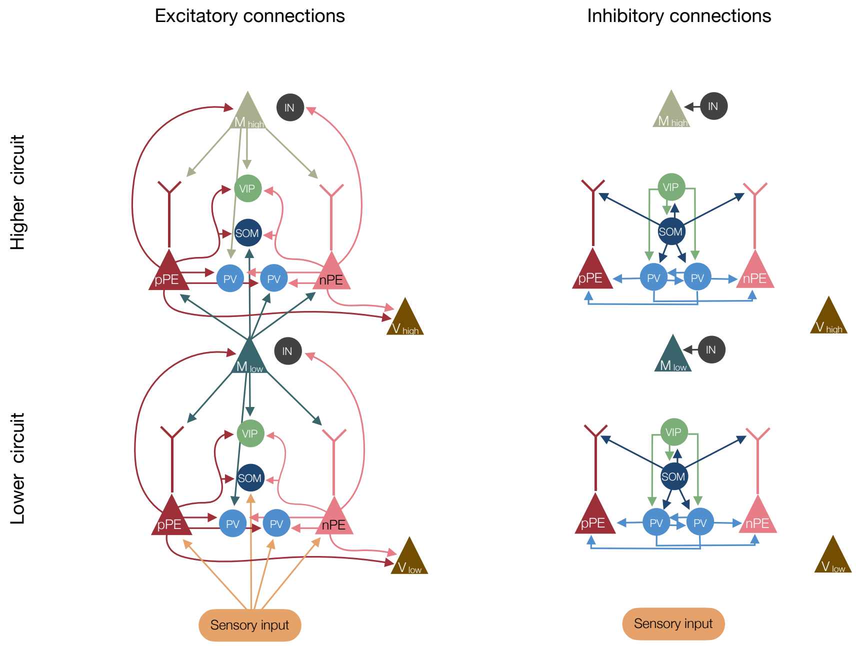

Supplementary Figure 1. Excitatory and Inhibitory connections in the model. Schematics of the full model with all cell types and the excitatory connections (left) as well as the inhibitory connections (right). Shown is the mean-field model in which the SOM cell receives the feedforward input and the VIP neuron receives the feedback projection from the respective M neuron. For Fig. 4 we also tested different mean-field models that only differ with respect to the inputs onto the SOM and VIP neuron.

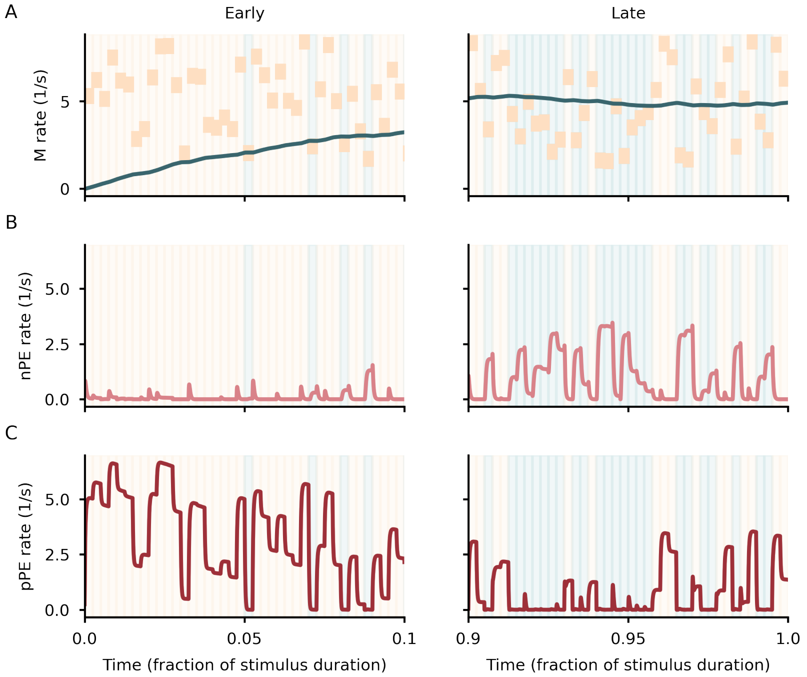

Supplementary Figure 2. nPE and pPE neurons drive the M neuron to encode the mean of the sensory input.
The M neuron (A) receives net inhibitory connections from the nPE neuron (B) and net excitatory connections from the pPE neuron (C). When the activity of the M neuron is below the sensory inputs (light orange shades in the background), the pPE neuron is strongly active and pushes the M neuron to increase. In contrast, when the activity of the M neuron is above the sensory inputs (light blue shades in the background), the nPE neuron is strongly active and pushes the M neuron to decrease. Hence, early in the process (first column), the pPE neuron is mainly active, while the nPE neuron is almost silent. Late in the process (second column), when the M neuron reaches the mean of the sensory stimuli, the number of times the nPE and pPE neurons are active is roughly balanced. Hence, the PE neurons constrain the M neuron and ensure that its activity does not drift too far from the mean.

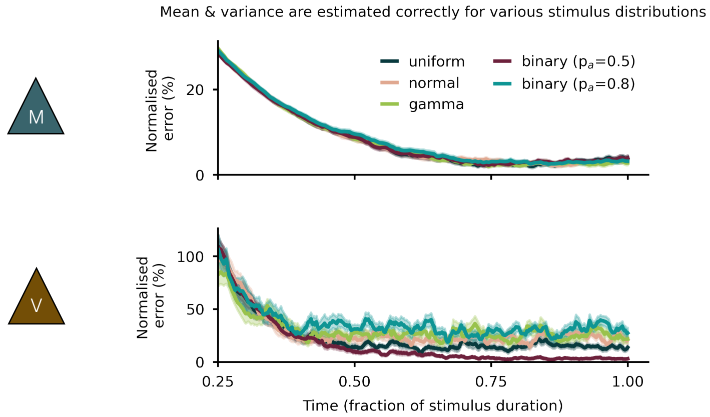

Supplementary Figure 3. Estimating mean and variance of different stimulus distributions.
Top: The normalised absolute difference between the averaged mean and the activity of the M neuron decreases to a near-zero level for all stimulus distributions tested. Bottom: The normalised absolute difference between the averaged variance and the activity of the V neuron decreases with small differences between the distributions tested. Parametrisation of the uniform distribution as in Fig. 2.

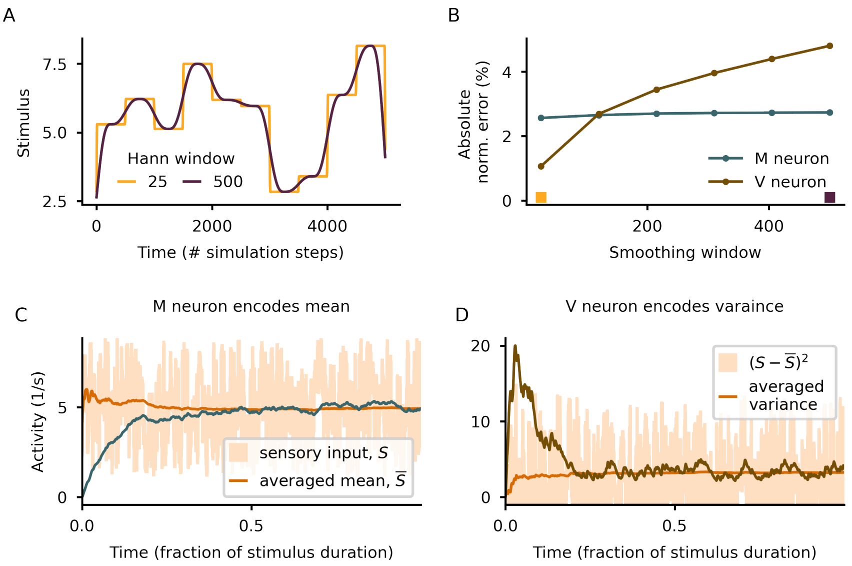

Supplementary Figure 4. Encoding the mean and the variance of continuously changing sensory stimuli. **(A)** The piecewise linear stimuli were smoothed by convoluting the signal with a Hann window of size 25 (yellow) or 500 (purple), producing continuously changing sensory stimuli (see Supplementary Methods for more details). **(B)** Normalized error for the difference between the M neuron's activity and the mean of the sensory stimuli (green), or for the difference between the V neuron's activity and the variance of the sensory stimuli (brown) with increasing Hann windows. The two examples depicted in A are denoted by the respective colored squares. The normalized errors are small for the range of Hann window's tested. **(C)** Example simulation for piecewise linear stimuli smoothed with a Hann window of 500. The M neuron's activity in the steady state resembles the mean of the stimuli. (D) Same as in C but for the variance. The V neuron's activity resembles the variance of the stimuli.

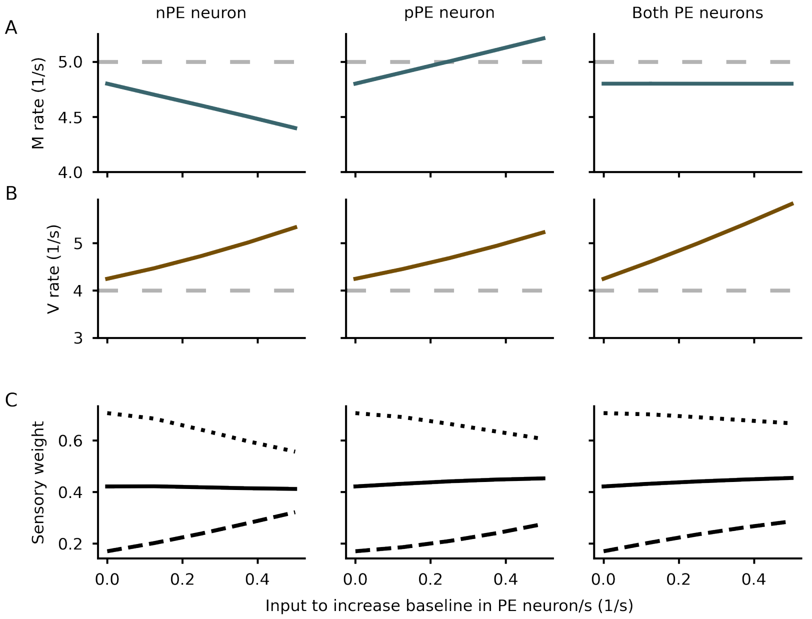

Supplementary Figure 5. Impact of non-zero baseline activity of nPE and pPE neurons. **(A)** The M neuron only encodes the mean of the feedforward input when the nPE and pPE neuron have similar baseline activities (third column). When the baseline activity of the nPE neuron is increased, the M neuron is biased toward values smaller than the actual mean of the sensory inputs (first column). When the baseline activity of the pPE neuron is increased, the M neuron is biased toward values larger than the actual mean of the sensory inputs (second column). True mean denoted by gray dashed lines. **(B)** The V neuron overestimates the variance of the feedforward input when the baseline of the PE neurons is increased. True variance denoted by gray dashed lines. **(C)** The sensory input is shifted towards 0.5 when the baseline activities of the nPE and/or pPE neuron are increased. That is, in a former sensory-driven regime (dotted lines), the dependence on the sensory inputs is slightly weakened. In a former prediction-driven regime (dashed lines), the dependence on the sensory inputs is slightly strengthened. Finally, in a regime in which sensory inputs and predictions are roughly equally weighted (solid lines), the changes do not have a pronounced effect.

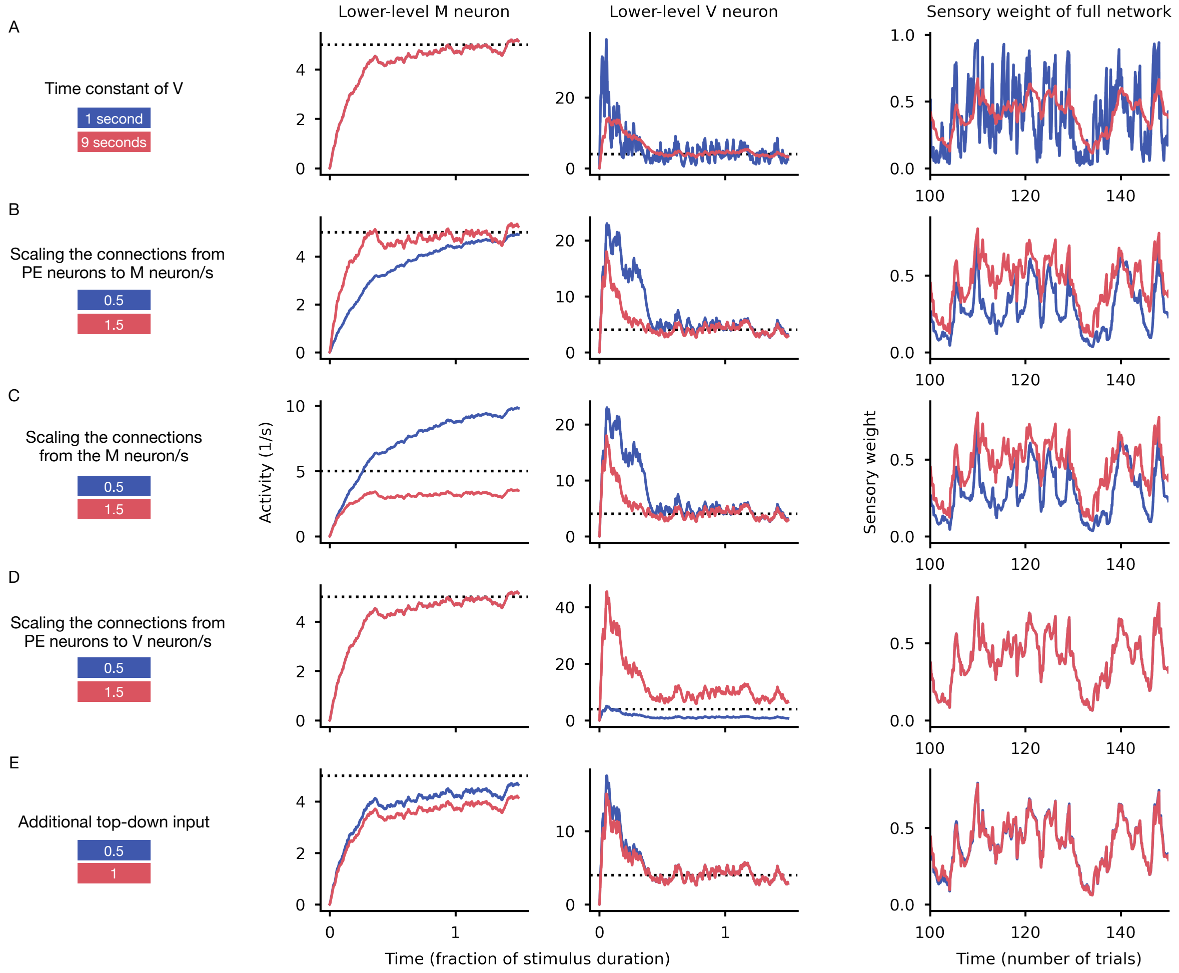

Supplementary Figure 6. **Analyzing network robustness.**
Changes in the connectivity to and from the M neurons (B and C), to the V neurons (D) and their rate time constants (A), or adding top-down input to the cells of the PE circuits (E) may alter the network's ability to encode the mean and the variance of the feedforward input which can lead to biased sensory weights. **(A)** Changing the time constant of the V neurons does not change the M neuron's ability to encode the mean nor the V neuron's ability to encode the variance. The estimate of the variance is, however, noisier the smaller the time constant, which is also reflected in the sensory weight that can vary strongly from trial to trial. **(B)** Scaling the connections from PE neurons to the M neuron does not affect the M neuron's ability to encode the mean but the speed at which the M neuron reaches the mean of the feedforward input. Hence, in the steady state, the V neuron encode the variance and the sensory weight is only slightly affected. **(C)** Scaling the outgoing connections from the lower-level M neuron biases its activity. However, the weighted activity of the lower-level M neuron (wEM * M) still encodes the mean of the feedforward input. The lower-level V neuron does encode the variance in the steady state. If the ongoing connections from the M neurons in both the lower-level and the higher-level circuit are scaled by the same amount, the sensory weight is almost unaffected. **(D)** Scaling the connections from PE neurons to the V neuron does not affect the M neuron's ability to encode the mean. However, the estimated variance, here encoded in the V neuron's activity, is shifted. Because this shift is present in both the lower and the higher-level V neuron, the sensory weight is not affected. **(E)** Adding top-down input to all neurons that receive the feedback projections from the respective M neurons biases the activity of the M neuron to values below the mean of the feedforward input (the difference is given by the top-down input). However, this does not change the variance estimation or the sensory weight because the PE neurons receive the same prediction as before, but here separated into the output of the M neuron and the top-down input. While the results in the second and third column were generated by simulating only the lower-level PE circuit, the results in the fourth column were generated by simulating the full network. See Supplementary Table 3 for parameters.

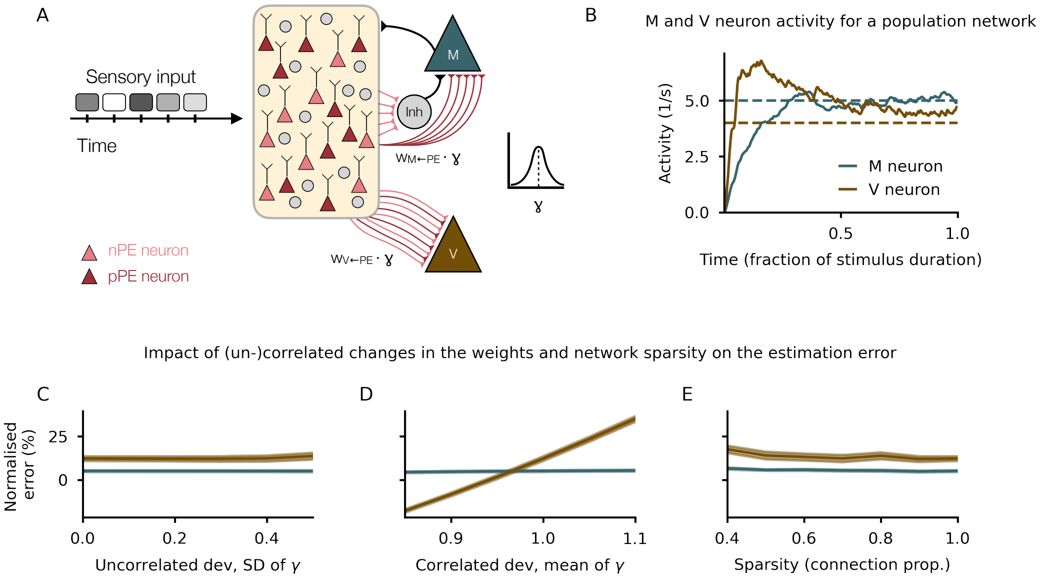

Supplementary Figure 7. Estimating mean and variance of sensory stimuli in a rate-based multi-cell population network.
(A) Illustration of the rate-based multi-cell population network and the stimuli over time. The weights from the PE neurons onto the M or V neuron are scaled by a factor γ drawn from a normal distribution $N(\mu_\gamma, \sigma_\gamma)$. **(B)** M and V neuron activities over time for one example parameterisation. **(C)** The normalised absolute difference between the averaged mean and the activity of the M neuron (dark green) or between the averaged variance and the activity of the V neuron (brown) for uncorrelated deviations (that is, increasing σ_γ). **(D)** same as in (C) but for correlated deviations (that is, increasing μ_γ). **(E)** same as in (C) but for network sparsity. To speed up simulations, we chose $\lambda^{\text{lower}} = 5 \cdot 10^{-2}$ for the rate-based multi-cell population network.

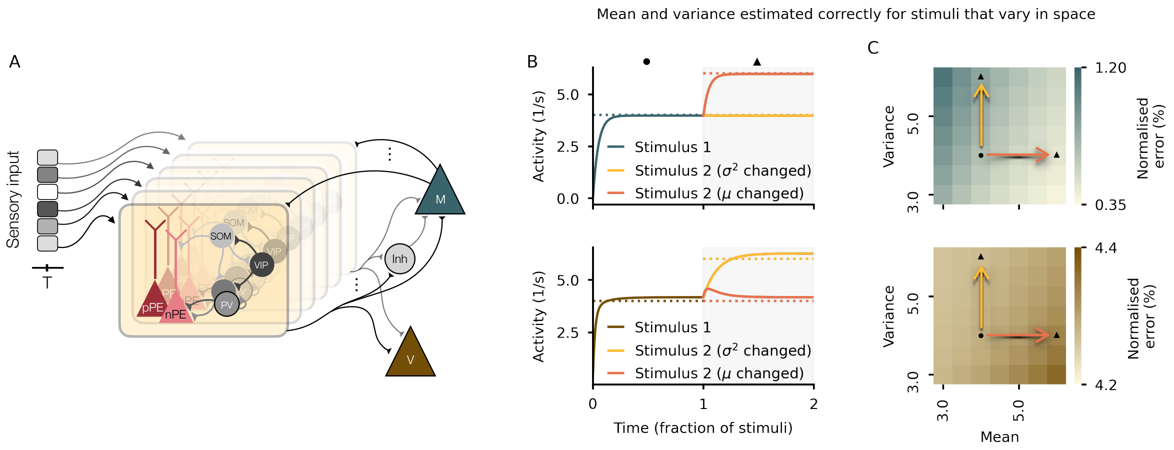

Supplementary Figure 8. Estimating mean and variance of spatial stimuli.
(A) Illustration of a network estimating the mean and variance of a stimulus that varies across space. To simulate selectivity, the network comprises 1000 identical, uncoupled mean-field networks each receiving a different input value drawn from a uniform distribution. **(B)** Activity of M neuron (top) and V neuron (bottom) for 2 stimuli. The second stimulus does either differ in the mean (orange) or the variance (yellow) from the first stimulus (indicated in C). **(C)** The normalised absolute difference between the averaged mean and the activity of the M neuron (dark green, top) or between the averaged variance and the activity of the V neuron (brown, bottom) for a range of different stimulus statistics. The examples from B are shown with colored arrows and markers. To speed up simulations, we chose $\lambda^{\text{lower}} = 3 \cdot 10^{-1}$.

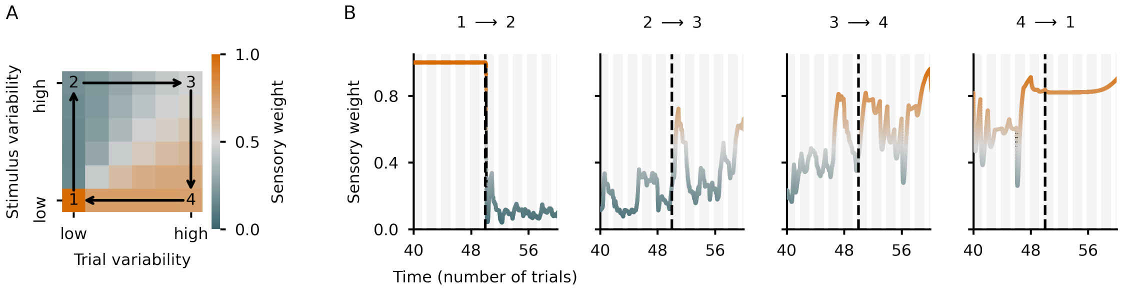

Supplementary Figure 9. Dynamic variance estimation allows flexible adaptation to changes in the stimulus statistics and environment.
(A) Illustration of the sensory weight for different input statistics. Numbers denote specific examples. Arrows denote the transitions between those statistics. **(B)** The sensory weight over time is shown for all transitions in (A). For the sake of clarity, we only show the trials 40-60. The switch to new input statistics occurs at trial 50. Parameters are listed in the Supplementary Methods.

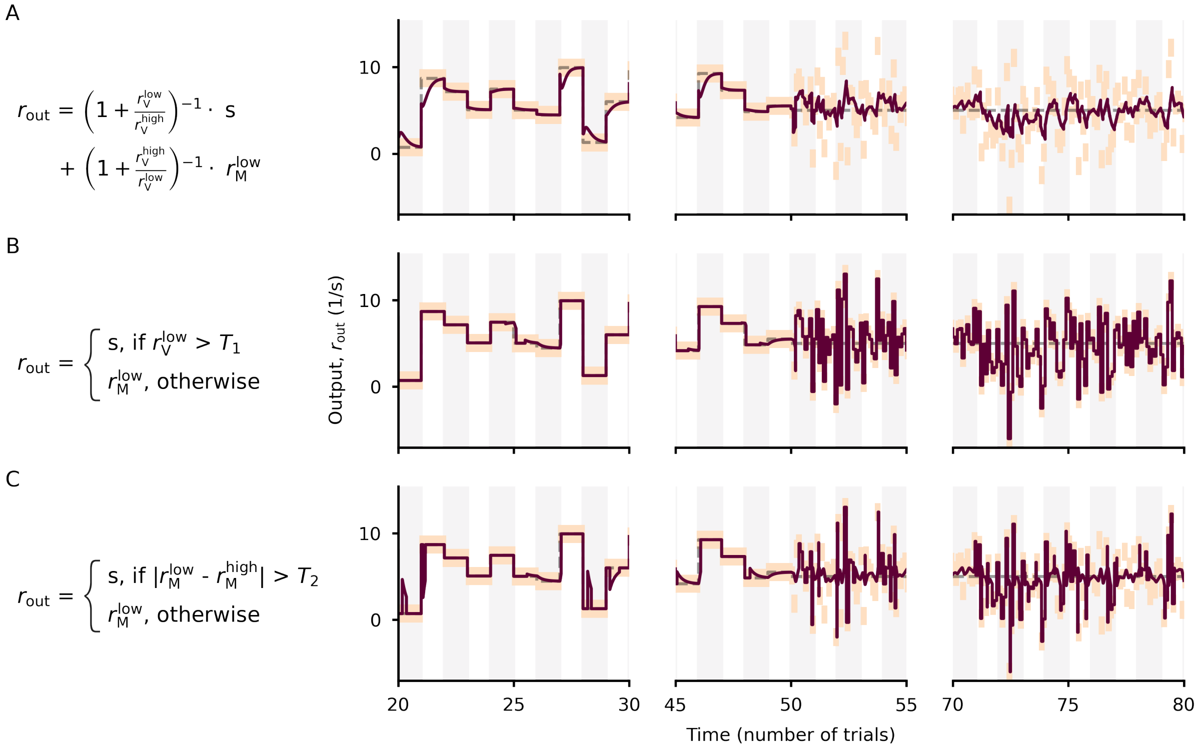

Supplementary Figure 10. Dynamic weighting of sensory input and prediction under different integration criteria.
(A) Sensory input (s) and prediction ($r_{\mathrm{M}}^{\mathrm{low}}$) are weighted according to their reliabilities (see Figure 3). Shown are the sensory input (orange) and the weighted output, r_{out} (dark brown) across three time windows (Left: trials 20–30, Middle: trials 45–55, Right: trials 70–80). Sensory input transitions from a zero-noise state (trials below 50) to a high-noise state (trials above 50). The dashed gray line represents the optimal output. **(B)** Same as (A), but the sensory input and prediction are combined based on the variance of the sensory input ($r_{\mathrm{V}}^{\mathrm{low}}$). When the variance is below a set threshold (here $T_1 = 1$), the output aligns with the prediction. Otherwise, it follows the sensory input. **(C)** Same as (A), but the sensory input and prediction are combined based on the similarity between the lower and higher M neurons ($r_{\mathrm{M}}^{\mathrm{low}}$ and $r_{\mathrm{M}}^{\mathrm{high}}$). If the absolute difference between the two is below a threshold (here $T_2 = 1$), the system relies on the prediction. Otherwise, it relies on the sensory input. While weighting based on reliabilities (A) does not perfectly track sensory input immediately after a change in the environment (μ) for the zero-noise condition, it performs adequately in the high-noise condition by effectively filtering out noise. In contrast, the approaches in (B) and (C) immediately track the sensory input in the zero-noise condition after a new trial but also closely follow the input in the high-noise state, thereby capturing the noise as well. This behavior can be mitigated by adjusting the thresholds (here $T_1 = T_2 = 1$). However, altering these parameters will reduce the accuracy of the output in the zero-noise condition.

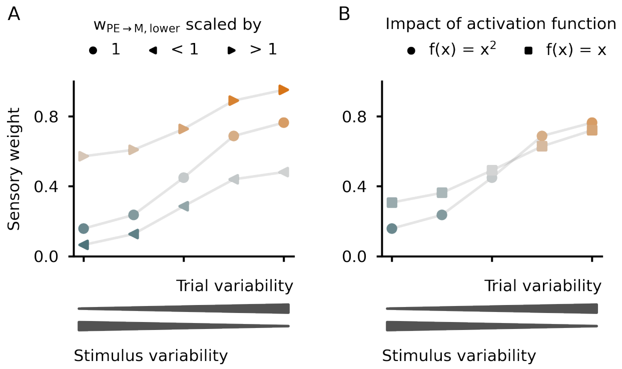

Supplementary Figure 11. Perturbing the weighting of sensory inputs and predictions by altering network properties.
(A) The weights from the PE neurons to the M neuron in the lower-order subnetwork are scaled by a factor 0.3 or 7, leading to a distorted sensory weight. If the update of the M neuron in the lower subnetwork is too slow (◄), the prediction is overrated. If the update of the M neuron in the lower subnetwork is too fast (►), the sensory input is overrated. **(B)** The precise activation function for the V neurons does not have a major impact on the sensory weight. Only for inputs with high stimulus variability, the sensory stimulus is slightly overrated when the quadratic activation function is replaced by a linear, rectified activation function.

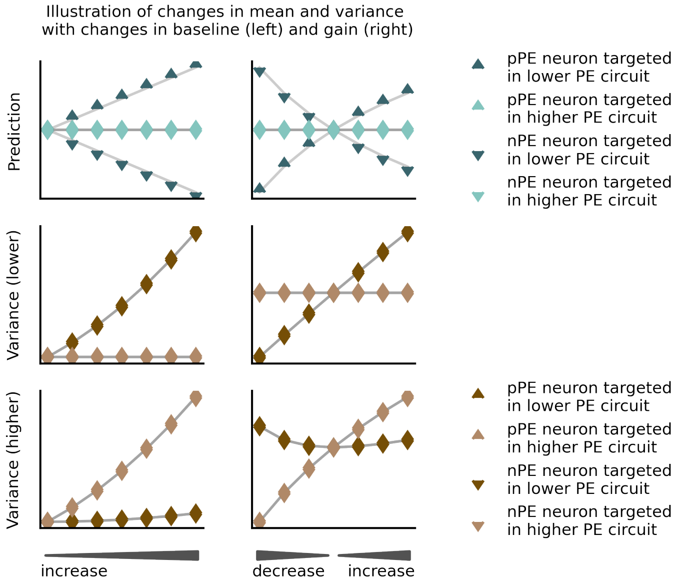

Supplementary Figure 12. Biased mean and variance estimation by changing the baseline and the gain of nPE and pPE. In a toy model, described in sections B.2 and B.3, the contribution of gain and baseline to the changes in the mean and variance estimation are summarized. The results shown are based on the Eqs. (18), (21), (23) and (26).

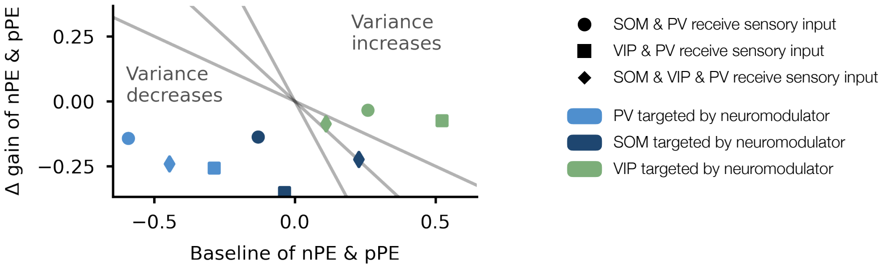

Supplementary Figure 13. The combined changes in baseline and gain of all PE neurons determine the shift in the sensory weight.
Whether and how a neuromodulator changes the sensory weight depends on the interneuron targeted and the effect this interneuron has on the baseline and gain of both PE neurons, which in turn does depend on the network it is embedded in. For small inputs, changes in the baseline dominate, while for large inputs, the changes in the gains dominate the shift in the sensory weight. Gray lines denote different mean inputs, illustrating that the same interneuron can decrease or increase the variance depending on the input regime.

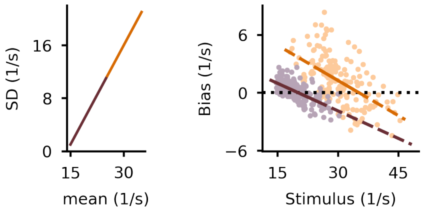

Supplementary Figure 14. Including scalar variability in the model
When scalar variability is included, that is, the stimulus standard deviation depends linearly on the stimulus mean, the bias is larger for stimuli drawn from the upper end of the stimulus distribution than from the lower end.

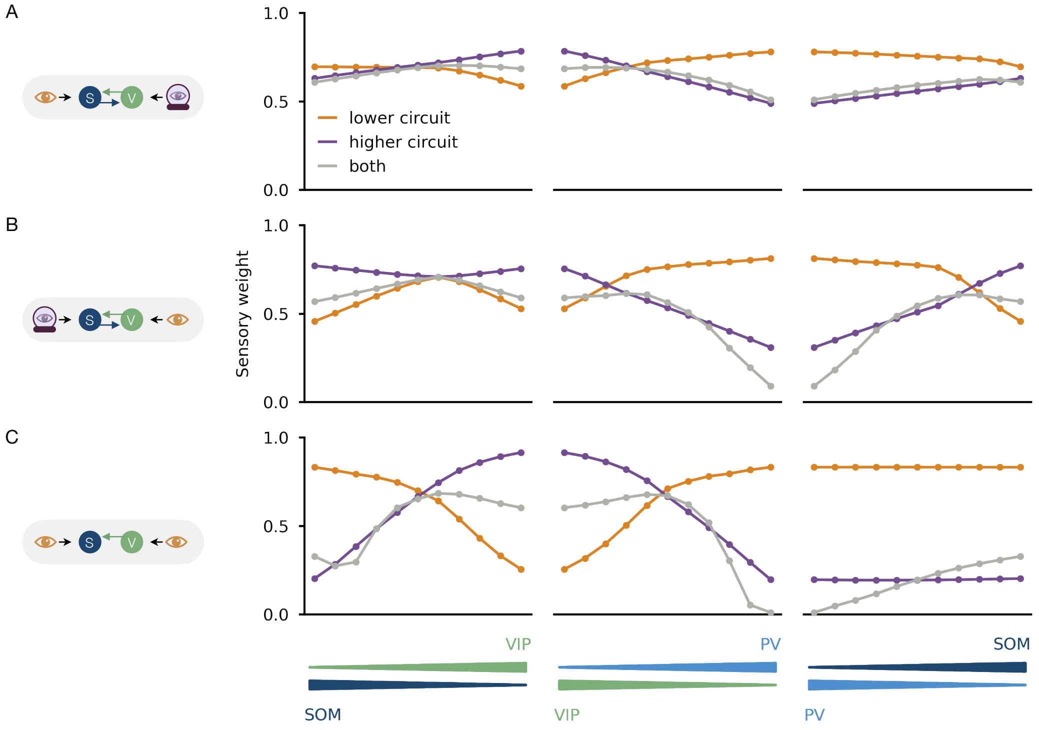

Supplementary Figure 15. The impact of neuromodulators acting on groups of interneurons in a sensory-driven input regime.
The sensory weight changes as a consequence of a neuromodulator acting on groups of interneurons either in the lower-level circuit (yellow), the higher level-circuit (purple) or both (grey) for three different mean-field networks (A: SOM neuron receives the feedforward input, while the VIP neuron receives the prediction thereof, B: VIP neuron receives the feedforward input, while the SOM neuron receives the prediction thereof, C: Both the SOM and the VIP neuron receive the feedforward input). Different combinations of interneurons that are simultaneously targeted by a neuromodulator are shown: SOM and VIP neurons (first column), VIP and PV neurons (second column), PV and SOM neurons (third column) targeted. The colored wedges/triangles indicate how much the respective interneuron was affected by the neuromodulator. In general, the changes in the sensory weight for a neuromodulator acting on interneurons in the higher-level PE circuit are the opposite of the changes seen for neuromodulators acting on the lower-level PE neurons. Before perturbation with a neuromodulator, the network was in a sensory-driven regime. Perturbation strength was 0.5/s.

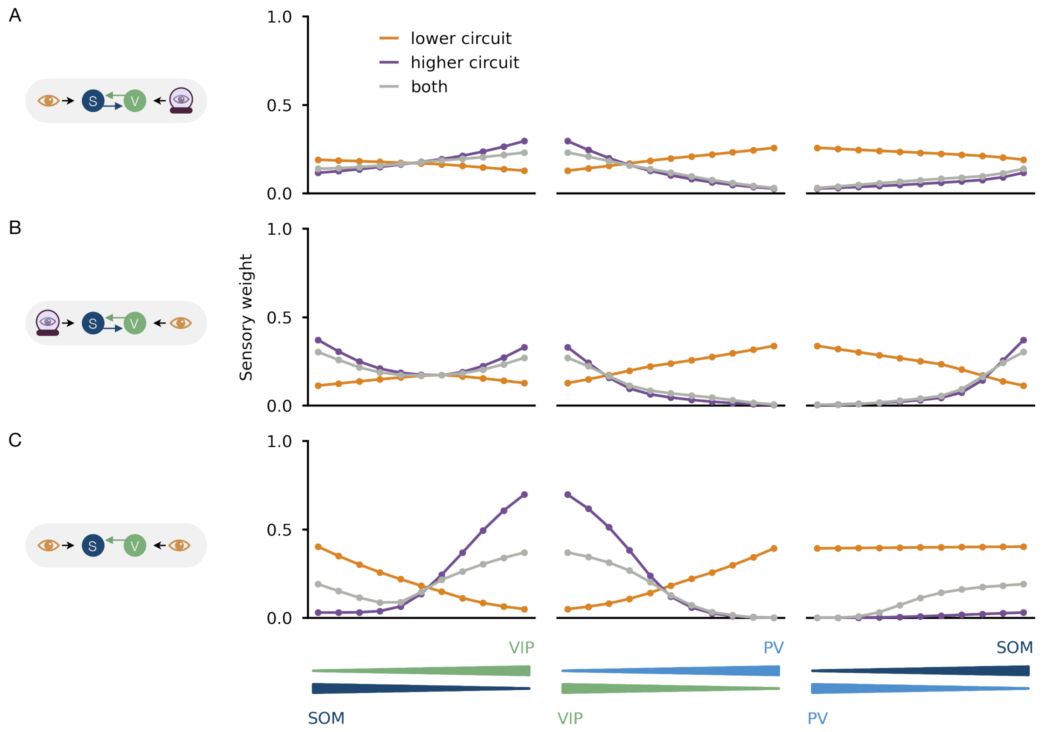

Supplementary Figure 16. The impact of neuromodulators acting on groups of interneurons in a prediction-driven input regime.
The sensory weight changes as a consequence of a neuromodulator acting on groups of interneurons either in the lower-level circuit (yellow), the higher level-circuit (purple) or both (grey) for three different mean-field networks (A: SOM neuron receives the feedforward input, while the VIP neuron receives the prediction thereof, B: VIP neuron receives the feedforward input, while the SOM neuron receives the prediction thereof, C: Both the SOM and the VIP neuron receive the feedforward input). Different combinations of interneurons that are simultaneously targeted by a neuromodulator are shown: SOM and VIP neurons (first column), VIP and PV neurons (second column), PV and SOM neurons (third column) targeted. The colored wedges/triangles indicate how much the respective interneuron was affected by the neuromodulator. In general, the changes in the sensory weight for a neuromodulator acting on interneurons in the higher-level PE circuit are the opposite of the changes seen for neuromodulators acting on the lower-level PE neurons. Before perturbation with a neuromodulator, the network was in a prediction-driven regime. Perturbation strength was 0.5/s.
