# Supplementary material for: Uncertainty estimation with prediction-error circuits
Source: Nat Commun. 2025 Mar 28;16:3036. doi: 10.1038/s41467-025-58311-6 (PMC11953419; doi:10.1038/s41467-025-58311-6)
Supplement: Supplementary file 2 — Description of Additional Supplementary Files [file 41467_2025_58311_MOESM2_ESM.pdf]

Description of Additional Supplementary Files

Supplementary Data 1: Local connectivity of the mean-field prediction-error circuit, as derived in Hertäg and Clopath (2022), where the SOM neuron is driven by feedforward sensory input, and the VIP neuron is driven by predictions.

Supplementary Data 2: Local connectivity of the mean-field prediction-error circuit, as derived in Hertäg and Clopath (2022), where the VIP neuron is driven by feedforward sensory input, and the SOM neuron is driven by predictions.

Supplementary Data 3: Local connectivity of the mean-field prediction-error circuit, as derived in Hertäg and Clopath (2022), where both SOM and VIP neurons are driven by the feedforward sensory input.

Supplementary Data 4: Local connectivity of the multi-cell prediction-error circuit, as derived in Hertäg and Clopath (2022).

Supplementary Data 5: Gain factors of excitatory cells in the multi-cell prediction-error circuit, as derived in Hertäg and Clopath (2022).
