# Supplementary material for: Uncertainty estimation with prediction-error circuits
Source: Nat Commun. 2025 Mar 28;16:3036. doi: 10.1038/s41467-025-58311-6 (PMC11953419; doi:10.1038/s41467-025-58311-6)
Supplement: Supplementary file 9 — Lasing Reporting Summary [file 41467_2025_58311_MOESM9_ESM.pdf]

nature research

Corresponding author(s): Loreen Hertäg

Lasing Reporting Summary

Nature Research wishes to improve the reproducibility of the work that we publish. This form is intended for publication with all accepted papers reporting claims of lasing and provides structure for consistency and transparency in reporting. Some list items might not apply to an individual manuscript, but all fields must be completed for clarity.

For further information on Nature Research policies, including our data availability policy, see Authors & Referees.

Experimental design

Please check: are the following details reported in the manuscript?

1. Threshold

Plots of device output power versus pump power over a wide range of values indicating a clear threshold

☐ Yes
☒ No

This is a computational modeling work, no experimental setup was used.

2. Linewidth narrowing

Plots of spectral power density for the emission at pump powers below, around, and above the lasing threshold, indicating a clear linewidth narrowing at threshold

☐ Yes
☒ No

This is a computational modeling work, no experimental setup was used.

Resolution of the spectrometer used to make spectral measurements

☐ Yes
☒ No

This is a computational modeling work, no experimental setup was used.

3. Coherent emission

Measurements of the coherence and/or polarization of the emission

☐ Yes
☒ No

This is a computational modeling work, no experimental setup was used.

4. Beam spatial profile

Image and/or measurement of the spatial shape and profile of the emission, showing a well-defined beam above threshold

☐ Yes
☒ No

This is a computational modeling work, no experimental setup was used.

5. Operating conditions

Description of the laser and pumping conditions
Continuous-wave, pulsed, temperature of operation

☐ Yes
☒ No

This is a computational modeling work, no experimental setup was used.

Threshold values provided as density values (e.g. W cm^{-2} or J cm^{-2}) taking into account the area of the device

☐ Yes
☒ No

This is a computational modeling work, no experimental setup was used.

6. Alternative explanations

Reasoning as to why alternative explanations have been ruled out as responsible for the emission characteristics
e.g. amplified spontaneous, directional scattering; modification of fluorescence spectrum by the cavity

☐ Yes
☒ No

This is a computational modeling work, no experimental setup was used.

7. Theoretical analysis

Theoretical analysis that ensures that the experimental values measured are realistic and reasonable
e.g. laser threshold, linewidth, cavity gain-loss, efficiency

☐ Yes
☒ No

This is a computational modeling work, no experimental setup was used.

8. Statistics

Number of devices fabricated and tested

☐ Yes
☒ No

This is a computational modeling work, no experimental setup was used.

Statistical analysis of the device performance and lifetime (time to failure)

☐ Yes
☒ No

This is a computational modeling work, no experimental setup was used.

March 2018
